# Response to: ‘A dose–response relationship between severity of disc degeneration and intervertebral disc height in the lumbosacral spine’—authors’ reply

**DOI:** 10.1186/s13075-016-0945-x

**Published:** 2016-02-11

**Authors:** Andrew J. Teichtahl, Donna M. Urquhart, Yuanyuan Wang, Anita E. Wluka, Stephane Heritier, Flavia M. Cicuttini

**Affiliations:** Department of Epidemiology and Preventive Medicine, School of Public Health and Preventive Medicine, Monash University, Alfred Hospital, 99 Commercial Road, Prahran, Melbourne, VIC 3004 Australia; Baker IDI Heart and Diabetes Institute, Commercial Road, Melbourne, VIC 3004 Australia

We welcome the comments from Emanuel et al. [1].

In a systematic review, Pfirrmann’s method [2] was endorsed as a valid and reliable ‘gold-standard’ for assessing intervertebral disc degeneration [3]. Nevertheless, the Pfirrmann system uses qualitative descriptors such as ‘disc height normal to slightly decreased’. This descriptor is highly subjective with confounding influences of gender, age and body habitus and the ambiguity of what represents a ‘normal’ disc height. There is no quantitative measure of disc height in the Pfirrmann grading system. This prompted us to validate disc height against the ‘gold-standard’.

In our work [4], we validate that for every one-grade increase in the Pfirrmann score, there is a reduction in disc height. For example, there was a 1.60 mm reduction (95 % confidence interval –2.37 to –0.83 mm) in disc height for each Pfirrmann grade increase at the level of L3/4, independent of age, gender, body mass index and smoking history. While Emanuel et al. highlight that disc height has diurnal and joint loading variation [1], the Pfirrmann Score has the same inherent limitations [2]. Any extraneous variability from such measures would have served to cause misclassification and reduced our chances of demonstrating significant results in the current study. Moreover, contemporaneous assessment of disc height and the Pfirrmann grade was made, mitigating extraneous variability. While there was large variability around the estimated marginal means, this is probably a reflection of the modest sample size (n = 72) and is accounted for in regression analyses, with highly significant results (all *p* ≤ 0.009).

In our study, we also demonstrated that disc height was smaller in people with high pain and/or disability, substantiating the clinical utility of the measure. While Emanuel et al. argue that factors such as high inter-subject and intra-subject variation limit the use of disc height as a clinical or epidemiological measure (e.g. from diurnal variation in water content of the disc or joint loading), this can be mitigated by standardising assessments to a particular time of the day (e.g. morning). While quantitative mapping shows promise, such techniques are highly sophisticated, expensive and not widely available.

We contend that our data validate disc height as a readily available, simple and effective means of assessing intervertebral disc degeneration, but do acknowledge that disc height may not be the best singular measure for disc degeneration. We welcome further efforts to identify continuous measures that sensitively assess disc degeneration.
